# Zinc oxide nanosphere for hydrogen sulfide scavenging and ferroptosis of colorectal cancer

**DOI:** 10.1186/s12951-021-01069-y

**Published:** 2021-11-27

**Authors:** Xiang Pan, Yuchen Qi, Zhen Du, Jian He, Sheng Yao, Wei Lu, Kefeng Ding, Min Zhou

**Affiliations:** 1 Department of Colorectal Surgery and Oncology, Key Laboratory of Cancer Prevention and Intervention, Ministry of Education, The Second Affiliated Hospital, Zhejiang University School of Medicine, Hangzhou, 310009 China; 2 Institute of Translational Medicine, Zhejiang University, Hangzhou, 310029 China; 3 State Key Laboratory of Modern Optical Instrumentations, Zhejiang University, Hangzhou, 310058 China; 4 Cancer Center, Zhejiang University, Hangzhou, 310058 China; 5 Laboratory of Gastroenterology, The Second Affiliated Hospital, Zhejiang University School of Medicine, Hangzhou, 310029 China; 6 The Cancer Hospital of the University of Chinese Academy of Sciences (Zhejiang Cancer Hospital), Institute of Basic Medicine and Cancer (IBMC), Chinese Academy of Sciences, Hangzhou, Zhejiang 310022 China

**Keywords:** Zinc oxide nanoparticles, Hydrogen sulfide, Colorectal cancer, Ferroptosis, Nanoengineering, Glutathione

## Abstract

**Background:**

Colorectal cancer is a common malignancy occurring in the digestive system and ranks second in cancer mortality worldwide. In colorectal cancer, hydrogen sulfide (H_2_S) is selectively upregulated, resulting in the further exacerbation of the disease. Therefore, the clearance of H_2_S and the regulation of the enzymes on the H_2_S pathways are of great significance for colorectal cancer therapy.

**Methods:**

Here, we investigated the H_2_S content in various clinical tumor tissues from patients and confirmed that overproduced concentration of H_2_S in colorectal cancer. Accordingly, we developed an H_2_S-responsive nanoplatform based on zinc oxide coated virus-like silica nanoparticles (VZnO) for the therapy of colorectal cancer.

**Results:**

Owing to its excellent H_2_S scavenging ability, VZnO could effectively reduce H_2_S content in colorectal cancer to prohibit the growth of CT26 and HCT116 colorectal cancer cells. Moreover, the removal of H_2_S in colorectal cancer also leads to tumor inhibition through activating ferroptosis, a non-apoptotic form of cell death. The biosafety-related toxicological and pathological analysis confirmed the low toxicity and high safety of VZnO in colorectal cancer treatment. Furthermore, as an H_2_S-responsible nanosystem, VZnO appears to have no therapeutic effect on other non H_2_S rich cancers, such as the 4T1 breast cancer model.

**Conclusions:**

We anticipate that the H_2_S-depletion-induced ferroptosis strategy using zinc oxide-based nanomaterials would provide insights in designing nanomedicines for colorectal cancer-target theranostics and may offer clinical promise.

**Graphic abstract:**

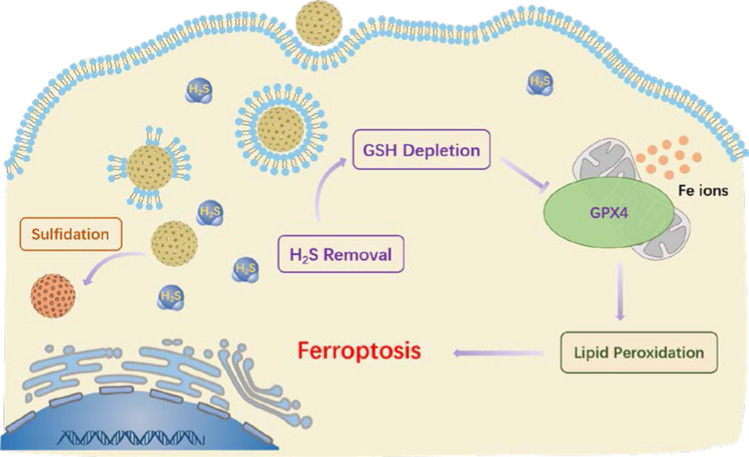

**Supplementary Information:**

The online version contains supplementary material available at 10.1186/s12951-021-01069-y.

## Background

Colorectal cancer (CRC), a common malignant tumor in the digestive system, ranks third in incidence and second in terms of mortality among the world. In 2020, more than 1.9 million new CRC cases and 935,000 deaths were estimated to occur, representing about one in ten cancer cases and deaths [1]. Conventional CRC treatments include endoscopic and surgical local excision, radiotherapy and systemic chemotherapy [2]. The use of these treatments tends to produce large side effects and poor prognosis, which seriously affects the quality of life of CRC patients [3]. Therefore, it is necessary to further study the mechanism of CRC and develop safe and effective methods for the CRC treatment.

Hydrogen sulfide (H_2_S) is a highly reactive endogenous gaseous signaling molecule that plays a crucial role in a variety of physiological functions [4], including activation of potassium channels [5], stimulation of kinase pathways [6], and inhibition of phosphodiesterase [7]. Recent studies have found that endogenous H_2_S is closely related to the occurrence and development of CRC. As the important parts of the digestive system, the H_2_S content in colorectal tissues tends to be higher than that in other normal tissues [8]. Due to the concentration dependence of H_2_S physiological effects, the increased H_2_S promotes the growth and progression of colorectal tumors, leading to higher mortality rates of CRC patients [9]. Classical research evidence demonstrated that cystathionine-β-synthase (CBS) is the key pathway for H_2_S production, and the up-regulation of CBS increases the content of endogenous H_2_S in the colorectal environment [10]. Therefore, using CBS inhibitors such as amino-oxyacetic acid (AOAA) can effectively reduce H_2_S content in CRC by down-regulating CBS, thus inhibiting the proliferation of CRC tumors [11]. However, CBS inhibitors represented by AOAA often affect multiple signaling pathways in the tumor, which is not conducive to the study of H_2_S and CRC. Therefore, finding a method that can directly remove the H_2_S in CRC is of great significance for the study of H_2_S and CRC.

Inspiringly, with the explosive development of nanotechnology, some traditional biomedical difficulties are being solved. The special size and structure of nanomaterials endow them with unique physical and chemical properties, and make them easier to concentrate at the tumor site [12–14]. Therefore, we believe that appropriate nanomaterials can be selected to directly eliminate H_2_S and thus be applied to the CRC treatment. In our previous study, a biocompatible nanosystem based on iron oxide-hydroxide nanospindles for magnetic resonance imaging (MRI) and H_2_S based reaction-enhanced combinational CRC treatment is developed [15]. Furthermore, Lin et.al reported that an H_2_S-activated Cu_2_O@CaCO_3_ nanostructure responding to the endogenous H_2_S and slightly acidic in the CRC microenvironment for CRC “turn-on” therapy [16]. These H_2_S scavenging nanomaterials, mainly based on the metal oxide family (Fe_3_O_4_ [17], Cu_2_O [18], MnO_2_ [19–21] and ZnO [22]), are expected to address the shortcomings of traditional CBS inhibitors because they act directly on endogenous H_2_S molecules. Using H_2_S to activate the diagnosis and therapy functions of intelligent theranostic agents has been considered an effective strategy to improve the treatment efficacy of CRC [16, 23]. However, the excessive functionality brings about the complexity of CRC research, and single-function nanomaterials that can effectively remove endogenous H_2_S are required to realize the research on H_2_S mediated CRC treatment.

**Scheme 1:**
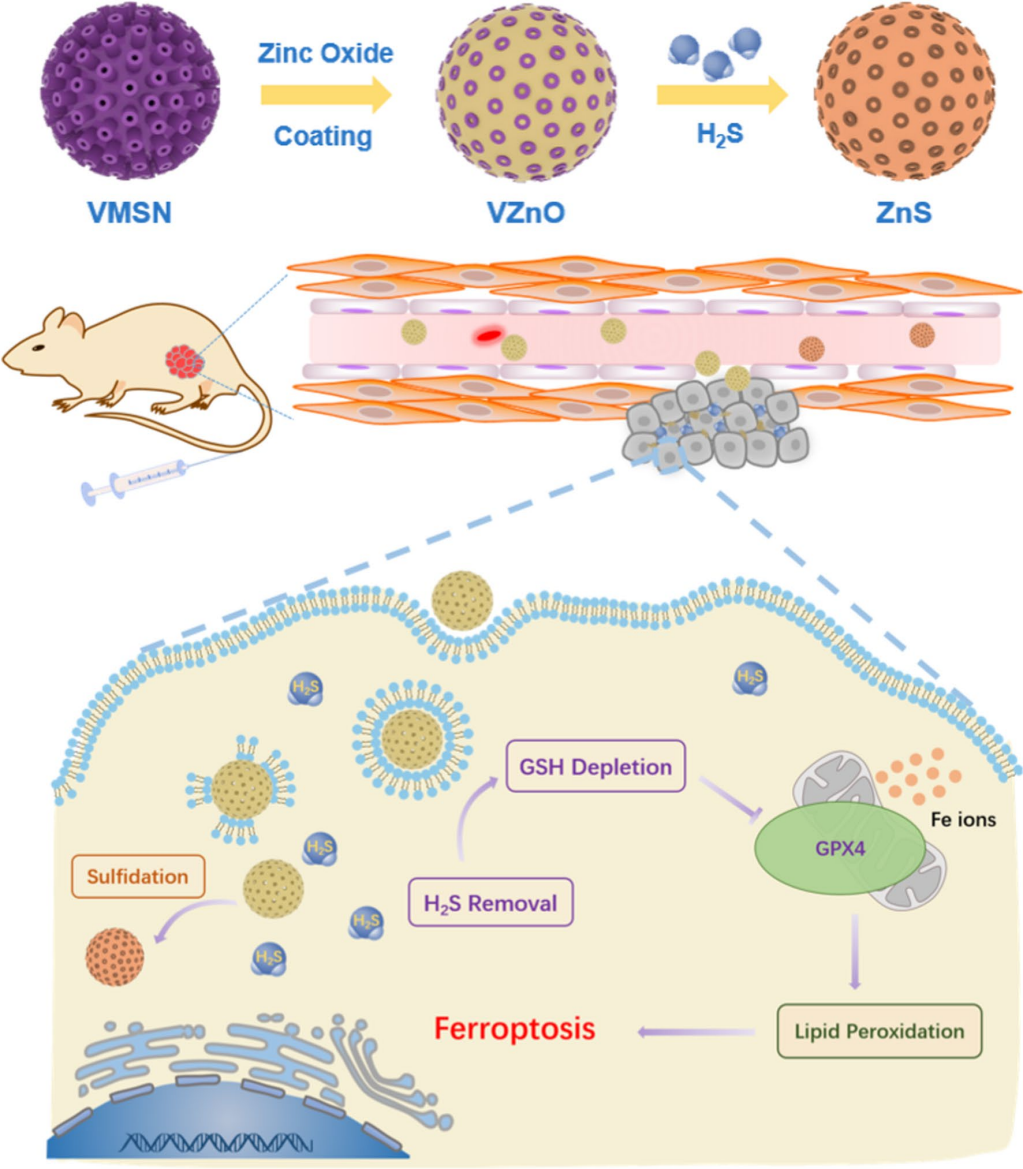
Schematic illustration of the synthetic route to VZnO and the H_2_S removal for the colorectal cancer therapy

Compared with other metal oxide desulfurizer, zinc oxide has outstanding desulfurization ability, high desulfurization accuracy, good stability, which makes it the most widely used desulfurizer in the industry [24]. Meanwhile, zinc oxide is relatively biocompatible and less toxic compared with other metal oxide nanoparticles, which further supports its potential biomedical applications [25]. Herein, we report an H_2_S-responsive nanoplatform based on zinc oxide coated virus-like silica (VZnO) nanoparticles for CRC therapy. We found that the decrease of H_2_S by VZnO leads to a significant decrease in intracellular glutathione (GSH) level, which eventually leads to ferroptosis in CRC cells. As a result, the VZnO nanoparticles could effectively suppress the growth of colorectal cancer (Scheme 1). Meanwhile, as an H_2_S-responsible nanosystem, VZnO appears to have no therapeutic effect on any other non H_2_S rich cancers, such as the 4T1 breast cancer model. Furthermore, the toxicological and pathological analysis confirmed the low toxicity and high safety of VZnO nanoparticles in CRC treatment. Our work may elucidate some of the mechanisms underlying H_2_S scavenging, ferroptosis and CRC, and provide an effective strategy for the CRC treatment.

## Materials and methods

### Human colorectal tissue samples

Human primary CRC and uninvolved colorectal from the identical resection specimens were obtained by surgical resection and stored at − 80 °C from 2018 to 2019 at The Second Affiliated Hospital, Zhejiang University School of Medicine. All experiments were approved by the Institutional Ethical Care and Use Committee of Zhejiang University. Informed consent was obtained from all patients, and experiments were performed in compliance with all relevant ethical regulations. No for in vivo animal study: AIRB-2021–952; Ref. No for in human dada: 2017–072.

### Materials

Tetraethyl orthosilicate (TEOS, 99%), hexamethylenetetramine (HMT, 99%) and zinc nitrate hexahydrate (Zn(NO_3_)_2_·6H_2_O, 99.95%), were purchased from Alfa Aesar Ltd. Hexadecyltrimethylammonium bromide (CTAB) and fluorescein isothiocyanate (FITC) were purchased from Sigma-Aldrich. NaOH, Na_2_CO_3_, cyclohexane, methanol and ethanol were supplied from the Beijing Chemical Reagent Company. All the chemical reagents were of analytical grade and used as received without further purification.

### Synthesis of zinc oxide coated virus-like silica nanoparticles

The virus-like mesoporous silica nanoparticles (VMSN) were synthesized according to the literature with slight modification. In brief, 0.8 mL of NaOH (0.1 M), 850 mg of CTAB and 60 mL of deionized water were added into a 100 mL round-bottom flask. The resulting solution was heated to 60 °C with constant stirring for 1.5 h. Then 4 mL of TEOS and 16 mL of cyclohexane were mixed and cautiously added to the flask to form an oil layer. The reaction was kept at 60 °C with constant stirring and then cooled down after 48 h for the following treatment. The as-obtained silica nanoparticles were separated by centrifugation. The product was extracted with 1 wt % solution of NaCl in methanol for 24 h, repeated three times, to remove the CTAB. The as-obtained silica nanoparticles were collected by centrifugation, washed with ethanol and water, and finally dried through lyophilization.

In a typical procedure, 100 mg VMSN, 230 mg of Zn(NO_3_)_2_·6H2O and 50 mL of deionized water were added into a 100 mL round-bottom flask. After well mixed, 140 mg of hexamethylenetetramine (HMT) was added to the aqueous dispersion under continuous stirring. The reaction was heated to 90 °C for 2 h and purified by centrifugation. The as-synthesized zinc oxide coated virus-like silica nanoparticles were washed with deionized water several times and dried through lyophilization, named VZnO.

### FITC loading and stability study

FITC was loaded on the VZnO by a soaking method. In brief, 20 mg of VZnO was wholly dispersed in 6 mL ethanol solution of FITC (0.5 mg/mL) and stirred overnight under dark conditions. The nanocomposites were collected by centrifugation, washed with ethanol and PBS for three times and dried via lyophilization. The product was denoted as VZnO@FITC. The stability of VZnO@FITC was researched by detecting the released amount of FITC with time. For determination, 10 mg of VZnO@FITC was redispersed in 20 mL of PBS buffer and stirred at 37 °C under dark conditions. After certain time intervals, 3 mL of the suspension was sucked out to centrifuge and the ultraviolet–visible (UV–vis) absorption of the supernatant was measured. The amounts of released FITC were calculated from the changes of the UV–vis absorbance.

### Characterization

TEM (Tecnai F20, FEI, USA) was applied to characterize the morphology and size of the nanoparticles. The phase and crystal structure of the nanoparticles were collected by an X-ray diffraction instrument (XRD, X’Pert PRO MPD, The Netherlands) operated at 40 mA and 40 kV using Cu Kα radiation. Dynamic light scatting (DLS) and Zeta potential analysis were provided by Zetasizer Nano-ZSE (Malvern Instruments, U.K.). UV–vis spectra were recorded by a UV-2600 spectrophotometer (Shimadzu, Kyoto, Japan). Fluorescence spectra were acquired with an RF-6000 fluorescence spectrophotometer (Shimadzu, Kyoto, Japan). Surface area and pore size were measured by Surface Area and Porosity Analyzer (AUTOSORB-IQ2-MP, QUANTACHROME). Fourier transform infrared (FT-IR) spectra were collected using the Thermo Nicolet NEXUS 470 FTIR spectrophotometer with the KBr pellet technique.

### Sulfidation process of the VZnO nanoparticles

NaHS was used to simulate endogenous hydrogen sulfide in the investigation of the sulfidation process of the VZnO nanoparticles. An aqueous dispersion of VZnO nanoparticles (35 mM, based on Zn element) was added to the NaHS solution (30 mM) at room temperature. After 2 h of incubation, the product was collected by centrifugation and washed twice with water. The obtained nanoparticles were characterized by X-ray diffraction (XRD), UV–vis and DLS.

### ***Assay of H***_***2***_***S generation***

To detect the generation of H_2_S in intestinal tissue of patients and mice, a unisense H_2_S microsensor (a miniaturized amperometric sensor with guard electrode) (Model H_2_S-MRCh, Unisense, Aarhus, Denmark) coupled to a unisense picoampere amplifier was used. 0.1 g Tissue (wet weight) was homogenized in 1 mL ice-cold potassium phosphate buffer (0.05 M). Adding 5 mL NaOH (1 M) and 5 mL 10% trichloroacetic acid to the central hole of the reaction bottle, the reaction bottle is blown 30 s by N_2_ before sealing. A thermostatic table concentrator initiated the reaction for 2 h at 37 °C. Finally, the reaction system was incubated at 37 °C for 2 h to terminate the reaction. The concentration of H_2_S in the solution was determined by the sensitive sulfur electrode method in the central hole. The rate of H_2_S formation was calculated.

### Western Blot

20 µg of protein extract was loaded on a 10% gel (PAGE Gel Fast Preparation Kit, EpiZyme-NP0321) and transferred to a polyvinylidene difluoride membrane. The membrane was incubated in 0.1% TBST with 5% non-fat dry milk (Blocker™ BLOTTO in TBS, Thermo-37530) for 2 h at room temperature. Primary antibodies were used at the following dilutions: rabbit-anti-GPX4 (Sigma-SAB4300725) 1:2000; rabbit-anti-CBS (Sigma-AV45746) 1:4000; rabbit-anti-COX2 (Sigma-SAB4200576) 1:5000; goat-anti-NOX1 (Sigma-SAB2501686) 1:5000; rabbit-anti-TRF1 (Sigma-SAB4502943) 1:500; rabbit-anti-GAPDH (Invitrogen-PA1-987) 1:3000; rabbit-anti-GGCT (Invitrogen-PA5-80,658) 1:1000; rabbit-anti-RRM1 (Invitrogen-PA5-75,989) 1:1000; rabbit-anti-RRM2 (Invitrogen-PA5-27,856) 1:1000. The membrane was washed with 0.1% TBST for 10 min (three times) at room temperature and followed by incubation in secondary antibody for 2 h at room temperature. Secondary antibodies: Goat anti-Rabbit IgG (Invitrogen-A31460), dilution 1:10,000; and Rabbit anti-Goat IgG (Invitrogen-A31402), dilution 1:10,000. The membrane was washed with 0.1% TBST for 10 min (three times) at room temperature. Antigen complexes were visualized and quantified with the Odyssey Infrared Imaging System (LI-COR).

### Immunohistochemistry

H&E stained tumor sections were stained using standard histological techniques. Immunohistochemistry was conducted using rabbit-anti-CBS (Sigma-AV45746) 1:4000. Last, slices were photographed with a Virtual slide microscope (Olympus VS120, Japan). The photographs were analyzed with the Image-Pro Plus 6.0 software (Media Cybernetics Inc., Silver Spring, MD, USA). Secondary antibodies: Goat-anti-rabbit IgG (Invitrogen-32460), dilution 1:60.

### Cell viability

The following cell lines were used in the study: HCT116, SW480, HT29 (all human colon cancer cells), MC38, CT26 (all mouse colon cancer cells), NCM460 (normal human colon mucosal epithelial cell), SKOV3 (human ovarian cancer cells), 4T1 (mouse breast cancer cells), MCF-10A (normal human breast epithelial cell), H1299 (human non-small cell lung cancer cell), B16F10 (murine melanoma cell). All the cell lines were purchased from American Type Culture Collection (ATCC). HCT116 were cultured in 1640 and CT26 were cultured in DMEM with 10% FBS and 1% antibiotics (100 U/mL penicillin and 100 μg/mL streptomycin) at 37 °C in a 5% CO_2_ atmosphere. 1 × 10^5^ CT26 and HCT116 were separately seeded in a 96-well plate and incubated with VZnO/ iron-dextrin (ID)/ ferrostatin-1 (Fer-1)/GSH at predetermined concentrations for 24 h. Cell viability was measured using the standard 3-(4,5-dimethylthiazol-2-yl)-2, 5-diphenyltetrazolium bromide (MTT) assay kit (YEASEN, Shanghai, China).

### Detection of reactive oxygen species

HCT116 and CT26 were seeded in 6-well plates at a density of 8 × 10^3^ cells per well and were incubated overnight, then treated with or without ID/VZnO for 24 h. Then the cells were stained with a DCFH-DA assay kit (YEASEN, Shanghai, China) or C-11 BODIPY (2 μM) and imaged under a fluorescence microscope (Zeiss, Oberkochen, Germany). The evaluation of generated reactive oxygen species (ROS) was further quantified by fluorescence microscope.

### Animal study

Balb/c athymic nude mice (5–6 weeks old) were purchased from the Shanghai SLAC Laboratory Animal Company. All animal experiments were approved by the Institutional Animal Care and Use Committee of Zhejiang University. To establish an orthotopic colon cancer model, CT26 transfected with luciferase (CT26/Luc) (2.0 × 10^6^ cells, 50 μL) in PBS were carefully injected into the cecal wall of mice under anesthesia by isoflurane (RWD Life Science). To establish an orthotopic breast model, 4T1 transfected with luciferase (4T1/Luc) (2.0 × 10^6^ cells, 50 μL) in PBS were carefully injected into the fourth pair of breast fat pads of mice. After a week, mice with CRC model were successfully established for fluorescence imaging. For mice toxicity studies in nude mice, mice were treated with VZnO/ID/PBS as described in the results. Mouse body weights were measured 2–3 times per week. At the end of the experiments, mice were euthanized, and blood and main organs were collected for analysis. The toxicity studies, including hematology, chemistry analyses, were performed by the Zhejiang Chinese Medical University Laboratory Animal Research Center.

### TEM analysis of tumor tissues

The sample was first fixed with 2.5% glutaraldehyde in phosphate buffer (0.1 M, pH 7.0) for more than 4 h; washed three times in the phosphate buffer for 15 min at each step; then postfixed with 1% OsO_4_ in phosphate buffer for 1–2 h and washed three times in the phosphate buffer (for 15 min at each step. Then the sample was first dehydrated by a graded series of ethanol (30%, 50%, 70%, 80%, 90% and 95%) for about 15 min at each step, then dehydrated by alcohol for 20 min. In the end, transferred to absolute acetone for 20 min. The specimen was placed in a 1:1 mixture of absolute acetone and the final Spurr resin mixture for 1 h at room temperature, then transferred to a 1:3 mixture of absolute acetone and the final resin mixture for 3 h, and ultimately placed in final Spurr resin mixture for overnight. Then, the specimen was transferred in Eppendorf containing Spurr resin and heated at 70 °C for more than 9 h. The specimen was sectioned in ultratome (LEICA-EM UC7) and sections were stained by uranyl acetate and alkaline lead citrate for 5–10 min respectively and observed in TEM (Hitachi Model H-7650).

### RNA-sequencing

The total RNA was extracted from HCT116 treated with VZnO/ID and control cells. The transcript was sequenced using the BGISEQ-500 sequencing platform (BGI Teeh Company, China). After rRNA depletion, the RNA fragments were reversely transcribed into cDNA by using an N6 random primer. The 5′ end of cDNA was phosphorylated, and 3′ end of cDNA was modified with stickiness “A”. Then, the cDNA was ligated with the adapter with sticky “T” at the 3′ end. Two specific primers are utilized to amplify the ligation DNA products. The DNA products were denatured by heat, and the single-stranded DNA was circularized by DNA ligase. Finally, the prepared DNA library was sequenced. Bowtie2 software was used to map clean reads to the reference gene, and HISAT software was utilized to map to the reference genome. Genes with a log2 fold change ≥ 1 were considered differentially expressed. The gene ontology analysis was carried out by the online program NetworkAnalyst (www.networkanalyst.ca).

### H&E staining

Murine-derived tumor tissue and main organs were embedded in optimal cutting temperature (OCT) compound (SAKURA-4583), flash frozen and kept at -80 °C until cutting. For H&E staining, slides were fixed in ice-cold acetone for 10 min, then immersed in filtered Harris modified Hematoxylin solution (Abcam-ab220365) for 5 min, then rinsed with tap water. The slides were dehydrated in ascending alcohol solutions (70%, 80%, 95%, and 100%) and cleared with xylene (Aladdin-1330–20-7) for 1 min each. Finally, the results of H&E staining were observed by a fluorescence microscope.

### Protein extraction from cells and tissues

For protein extraction from cells, cell pellets were lysed buffer (Beyotime-P0013B) after rinsed by pre-cooling PBS, for 30 min at 4 °C on a rotary shaker. For protein extraction from intact or decellularized tissues, 0.5 g of freshly dissected tissue was disrupted at 4 °C using a homogenizer. Protein concentration was determined using the BCA protein assay kit (23225; Thermo Fisher Scientific). All protein suspensions were kept at 0 °C until use.

### Glutathione detection

GSH was measured by using a Total Glutathione Assay Kit (Beyotime Institute of Biotechnology) according to the manufacturer's protocol. Absorbance was measured at 412 nm and the result was calculated as nmol/mg protein.

### Fluorescence microscopy examination of lipid peroxidation with C11-BODIPY

Before the experiment, cells were seeded in a confocal culture dish (2 × 105 cells per dish). After incubation for 24 h, the old medium was aspirated, followed by the addition of fresh culture medium. After another 6 h incubation, the culture medium was aspirated, and the resulting cells were washed twice with PBS. Next, MCF-7 were stained with 1 mL of C11-BODIPY (10 μM) at 37 °C for 20 min. Finally, the resulting cells were washed again with PBS 3 times, and further observed under confocal fluorescence microscopy.

### Statistical analysis

Results are expressed as the mean ± standard deviation unless otherwise stated. Statistical differences among experimental groups were analyzed by Student's *t*-test. *P* < 0.05 was considered statistically significant. All statistical calculations were performed using GraphPad Prism 6, including assumptions of tests used (GraphPad Software Inc, CA, USA).

## Results and discussion

### ***The overproduction of H***_***2***_***S in human CRC***

**Fig. 1:**
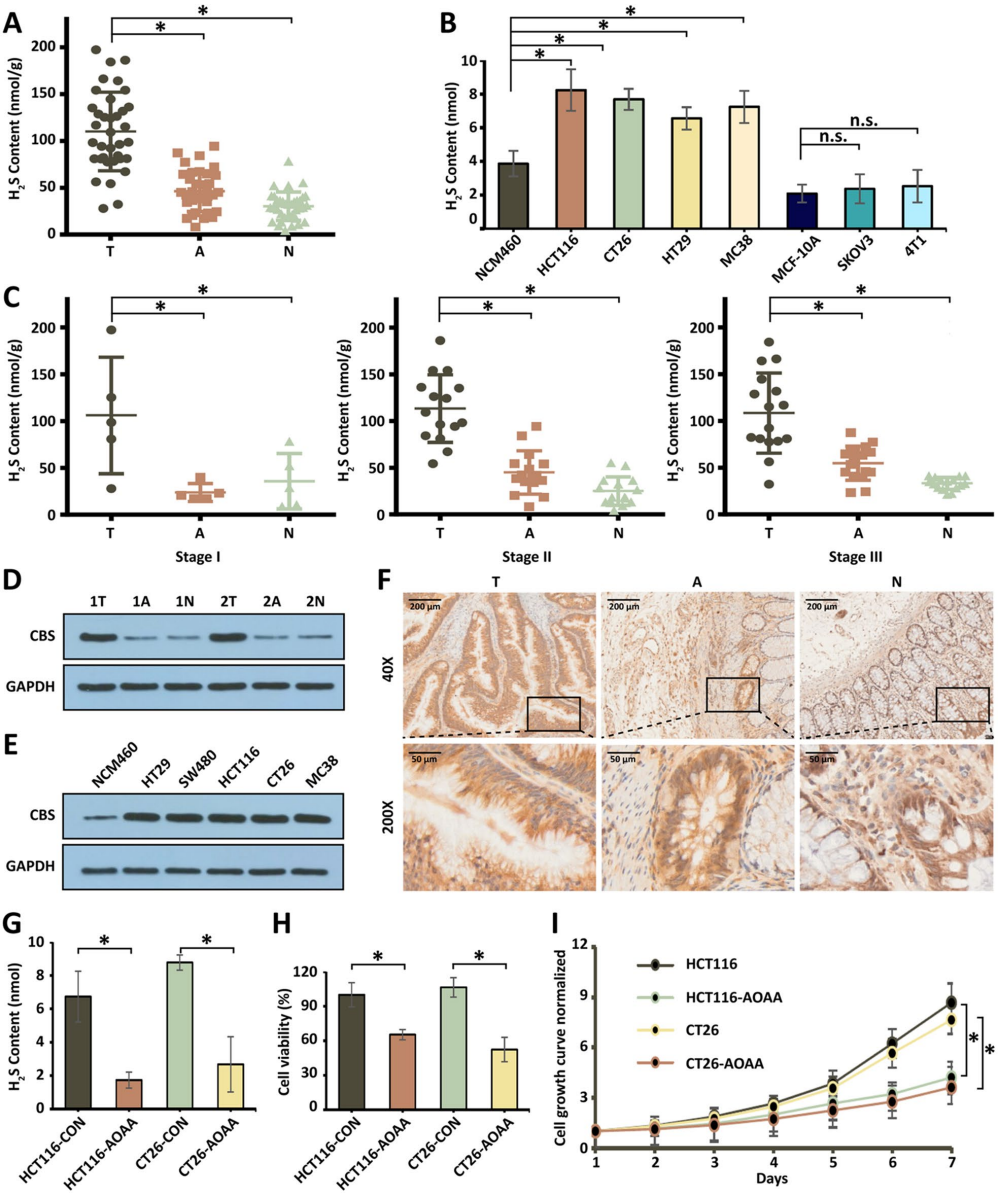
The overproduction of H_2_S in human CRC. **A**, **B** H_2_S production measured in human colorectal cancer specimens (*P < 0.05 vs. normal mucosa) and in colorectal cancer cell lines (*P < 0.05 vs. NCM460 cells; n.s.: no significant difference v.s. MCF-10A) by the H_2_S microsensor. (T: tumor tissue; A: aside colorectal tumor tissue; N: normal colorectal tissue). **C** H_2_S production in different stage of human colorectal cancer specimens measured by the H_2_S microsensor. **D** Western Blot of CBS protein expression in human colorectal cancer specimens, paired with the adjacent tissue and corresponding normal mucosa tissues. Membranes are re-probed for GAPDH expression to show that similar amounts of protein were loaded in each lane. **E** Immunohistochemistry of CBS protein expression in human colorectal cancer specimens, paired with the adjacent tissue and corresponding normal mucosa tissues. **F** Western Blot of CBS protein expression in colon cancer cell lines (vs. NCM460). **G** H_2_S production is measured in colon cancer cell lines treated by AOAA. **H** Cell viability of the HCT116 and CT26 treated with AOAA for 24 h. **I** Cell growth of the HCT116 and CT26 treated with AOAA within 6 days. (*P < 0.05), (n.s.: No significant difference, *P* value > 0.05)

It has been reported that H_2_S plays a vital role in inflammatory response and CRC development [26–29]. To explore the role of H_2_S in CRC, we first examined the concentration of H_2_S in human CRC specimens (Additional file 1: Table S1) and several typical CRC cells (HCT116, CT26, HT29, and MC38). As shown in Fig. 1A, the specific expression of H_2_S in CRC human specimens was significantly higher than that in normal colorectal tissues and aside colorectal tumor tissues. Furthermore, H_2_S is obviously overproduced in CRC cells compared to non-CRC cell lines, including MCF-10A normal human breast epithelial cells, SKOV3 ovarian cancer cells, and 4T1 breast cancer cells (Fig. 1B). We then found that H_2_S was consistently overexpressed at different stages of CRC development (Fig. 1C), and there was no significant difference in H_2_S expression between different stages of CRC patients (Additional file 1: Fig. S1). It is worth noting that the content of H_2_S in normal human colorectal mucosal epithelial cells is significantly higher than that in non-colon cell lines (Fig. S2). Since endogenous H_2_S in CRC is mainly produced from CBS [13], we studied the up-regulation of CBS in human CRC specimens compared with the patient-matched normal colorectal tissue by Western Blot and Immunohistochemistry (Fig. 1D, E). Similar CBS expression trends were also observed in normal colon cells and colon cancer cell lines (Fig. 1F). Then we use AOAA, a hydrogen sulfide synthase inhibitor, to block the H_2_S-producing activity of CBS in HCT116 and CT26 (Fig. 1G). After the treatment of AOAA, both cell viability (Fig. 1H) and cell growth (Fig. 1I and Additional file 1: Fig S3) of HCT116 and CT26 demonstrated significant inhibiting effects.

### Preparation and characterization of VZnO

In addition to CBS-derived, H_2_S is mainly a product of cysteine metabolism among many compounds produced by intestinal microorganisms in the metabolic activities of amino acids, which has exerted influences on the metabolism, physiology and physiological pathology of the host colonic mucosa [30]. Mesoporous materials have attracted extensive research interest because of their large specific surface area, large pore volume, uniform and orderly mesopores, and rich skeleton structure [31]. Taking advantage of the excellent ability of zinc oxide to remove hydrogen sulfide, we decided to develop an H_2_S-responsive VZnO nanoplatform for CRC therapy.

**Fig. 2:**
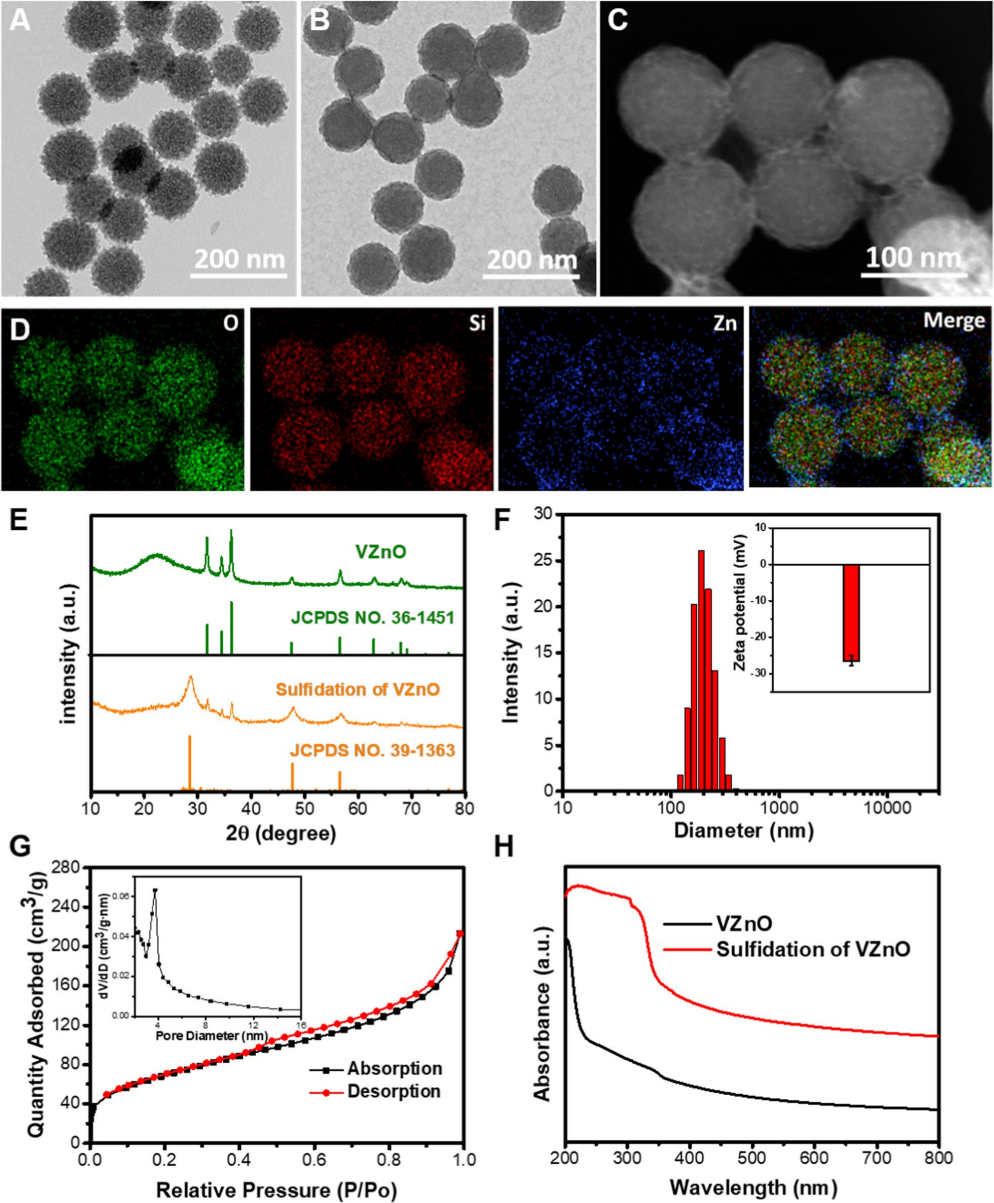
Preparation and characterization of VZnO. **A**, **B** TEM images of original VMSN template and VZnO nanoparticles, respectively. **C**, **D** HAADF-STEM image and corresponding elemental mapping of VZnO nanoparticles. **E** XRD patterns of the VZnO nanoparticles before and after sulfidation reaction, respectively. **F** Size distribution of the VZnO from DLS analysis. Inset: zeta potential of the nanoparticles. **G** Nitrogen adsorption–desorption curves of the VZnO nanoparticles. Inset: Pore-size distribution curve of the VZnO nanoparticles. **H** UV–vis absorption spectrum of VZnO nanoparticles before and after sulfidation reaction, respectively

The synthetic route of the VZnO nanosphere was illustrated in Scheme 1. Firstly, the virus-like mesoporous silica nanoparticles with monodispersed size (≈120 nm) and surrounding surface morphology were synthesized as templates (Fig. 2A, Additional file 1: Fig. S4). Then, the zinc oxide layer was successfully deposited in situ on the surface of VMSN, leading to the formation of VMSN@ZnO nanoparticles (Fig. 2B, C). The elemental mapping images showed that Si and O elements were homogeneously distributed in the core of the VZnO nanoparticles, while the Zn element was homogeneously distributed in the shell of the nanoparticles (Fig. 2D). The XRD patterns confirmed that the crystal structure of the VZnO nanoparticles agreed well with the standard zinc oxide (JCPDS No. 036–1451) (Fig. 2E). In addition, DLS studies revealed a monodisperse size distribution for VZnO with an average diameter of 203 nm (Fig. 2F). Nitrogen adsorption − desorption experiments showed that VZnO has a Brunauer − Emmett − Teller (BET) surface area of 241 m^2^g^−1^ and a median pore size of 3.23 nm (Fig. 2G). The mesoporous structure and negative Zeta potential make VZnO an excellent drug carrier. Here, FITC was loaded into the VZnO by a soaking method, yielding VZnO@FITC for the real-time fluorescence imaging of the nanoparticles (Additional file 1: Fig. S5).

### Sulfidation of VZnO

As a classical desulfurized, zinc oxide exhibits outstanding H_2_S scavenging ability. The chemical reaction between zinc oxide and hydrogen sulfide is illustrated by the following equation.

1 $${\text{ZnO}}\left( {\text{s}} \right){\text{ + H}}_{{2}} {\text{S}}\left( {\text{g}} \right) \, \to {\text{ ZnS}}\left( {\text{s}} \right){\text{ + H}}_{{2}} {\text{O}}\left( {\text{g}} \right)$$

zinc oxide can react with surrounding gaseous H_2_S efficiently to consumes H_2_S while generates zinc sulfide as the product. [32, 33]. Thus, the ingredient of VZnO before and after the sulfuration reaction was confirmed first. To simplify the research work, NaHS was chosen as the sulfur source to simulate the endogenous H_2_S gas. After the reaction with NaHS, the XRD patterns of VZnO showed prominent zinc oxide peaks disappear and zinc sulfide peaks appear, indicating that VZnO was vulcanized into ZnS (Fig. 2E). Meanwhile, the UV–vis absorption of VZnO increased significantly within 350 nm after the sulfuration reaction process (Fig. 2H). With the VMSN as the core, the morphology of VZnO did not change significantly after a short time sulfidation reaction (Additional file 1: Fig S6). The above results indicate that VZnO reacts with H_2_S to form ZnS. The above results indicate that VZnO exhibits outstanding H_2_S reactivity, making it a promising candidate for CRC therapy.

### ***The H***_***2***_***S scavenging of VZnO in CRC***

**Fig. 3:**
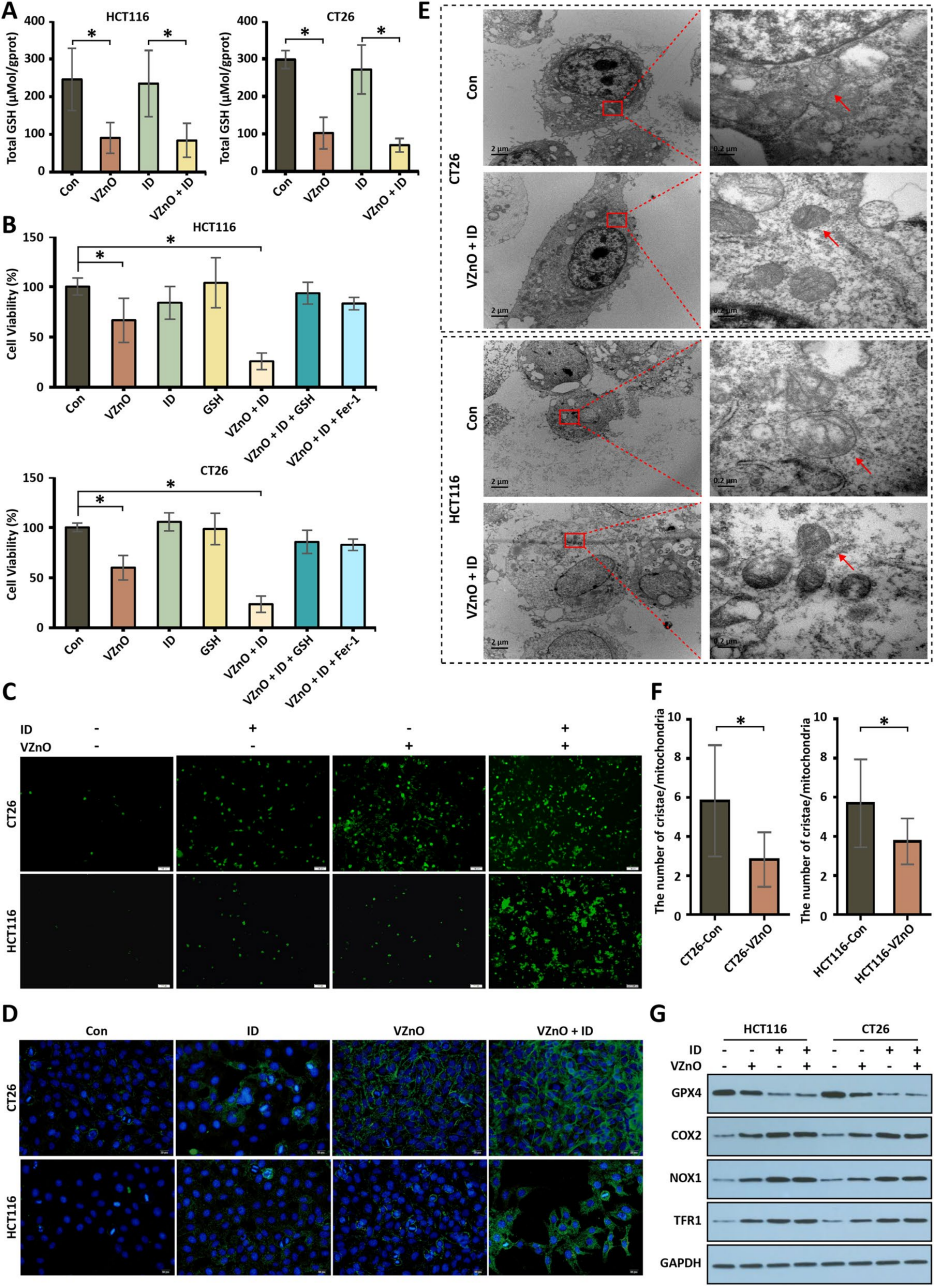
The H_2_S scavenging and tumor ferroptosis induced by VZnO in CRC. **A** The GSH detected by total glutathione assay kit in HCT116 and CT26 after VZnO and ID treatments. **B** Cell viability by MTT assay in HCT116 and CT26 after VZnO, ID, GSH and Fer-1 treated. **C** Intracellular ROS levels measured by DCFH-DA assay kit in HCT116 and CT26 with or without VZnO and ID treated. **D** Colocalization of oxidized lipid and nucleus co-stained by BODIPY C11(green) and DAPI (blue) in HCT116 and CT26 cells. **E** Representative picture of the ultrastructure of mitochondria in HCT116 and CT26 after VZnO and ID treated. **F** Average number of mesenchymal cristae per mitochondrion in VZnO-treated cells (statistics of 20 mitochondria) **G** Representative GPX4, COX2, NOX1 and TFR1 protein expression by Western Blot in HCT116 and CT26 with or without VZnO and ID treated. Membranes were re-probed for GAPDH expression to show that similar amounts of protein were loaded in each lane. (*P < 0.05), (n.s.: No significant difference, *P* value > 0.05)

It has been reported that H_2_S plays an important role in GSH biosynthesis [34, 35]. Therefore, we studied the effect of H_2_S clearance on CRC cells by studying the changes of GSH. GSH is a major intracellular antioxidant factor that inhibits ROS-dependent p38/MAPK activation by reducing ROS levels [23]. According to the report, GSH and GSH metabolizing enzymes are present at elevated levels in colonic tumors [36–38]. However, the regulatory mechanism of H_2_S on glutathione in human CRC cell lines and tissues remains unelucidated. At first, we detected the total GSH levels in HCT116 and CT26 after VZnO treatment for 24 h. GSH levels in HCT116 and CT26 are significantly reduced after H_2_S is removed (Fig. 3A). Following treatment with VZnO for 24 h, the cell viability of both the HCT116 and CT26 was decreased via the MTT based cell viability assay. Moreover, the rescue experiment showed that GSH reversed the VZnO-induced decline in cell viability (Fig. 3B). And we evaluated the effect of VZnO on cell viability of 4T1 or MCF-10A or SKOV3 from 24 h. We did not observe a significant decrease of cell viability 24 h after treatment, indicating the negligible cytotoxicity of VZnO. (Additional file 1: Fig. S7).

GSH deficiency, an essential antioxidant in mammalian cells, leads to an increase in intracellular ROS. Then, we elevated ROS levels in HCT116 and CT26 after 24 h treatment with VZnO (Fig. 3C). We found that VZnO can reduce the total intracellular GSH content by scavenging H_2_S.

### The ferroptosis of cancer cells

After GSH depletion, glutathione peroxidase 4 (GPX4) activity decreases, and lipid oxides cannot be metabolized by GSH reductase catalyzed by GPX4, ultimately contributing to ferroptosis [39, 40]. GSH provides electrons to the critical regulator of ferroptosis, GPX4, which is one of the most efficient enzymes for reducing lipid peroxides (–OOH) in membranes to their corresponding alcohols (–OH) [41, 42]. The occurrence of ferroptosis is always accompanied by a series of changes at the cellular, molecular, and genetic levels similar and different from other forms of cell death [43, 44]. Ferroptosis phenotypes were determined primarily by observing the iron-dependent accumulation of ROS combined with morphological changes at the cellular and subcellular levels [45, 46]. Evaluation of molecules associated with ferroptosis, such as labile iron, ROS, and glutathione, provides a means of monitoring the process of ferroptosis in vivo and *in* vitro. Protein and genetic analysis led to a deeper understanding of ferroptosis [47, 48]. Ferroptosis is associated with dramatic morphological changes of mitochondria, including mitochondrial fragmentation and decreased number of cristae [49, 50]. In this part, we used some experiments that focus on the characteristics and biomarkers of ferroptosis.

To further verify that ferroptosis occurred, we found that the cell viability of HCT116 and CT26 was further decreased treated with VZnO and ID (100 μM) (Fig. 3B). Moreover, after HCT116 and CT26 were treated with VZnO and ID for 24 h, a DCFH-DA assay kit was used to detect intracellular ROS levels. Green fluorescence was significantly enhanced, indicating that intracellular ROS levels were increased (Fig. 3C). Using the fluorescent probe C11-BODIPY, we observed a significant increase in lipid oxidation prior to cell death in HCT116 and CT26 treated with VZnO and ID for 24 h (Fig. 3D). TEM images (Fig. 3E) revealed structural aberrations of mitochondria (the red arrow in Fig. 3E) in malignant epithelial cells, including mitochondria smaller than normal cells and mitochondrial membrane shrinkage, as well as reduction and disappearance of mitochondrial cristae, even without impaired membrane integrity (Fig. 3F). Reduced GPX4 levels and enhanced expression levels of NOX1, COX2 and TFR1 were observed in HCT116 and CT26 treated with VZnO and ID for 24 h, suggesting ferroptosis is activated (Fig. 3G). These all indicated that ferroptosis was activated in HCT116 and CT26 after H_2_S was removed by VZnO and ID.

### In vivo and ex vivo biodistribution of VZnO

**Fig. 4:**
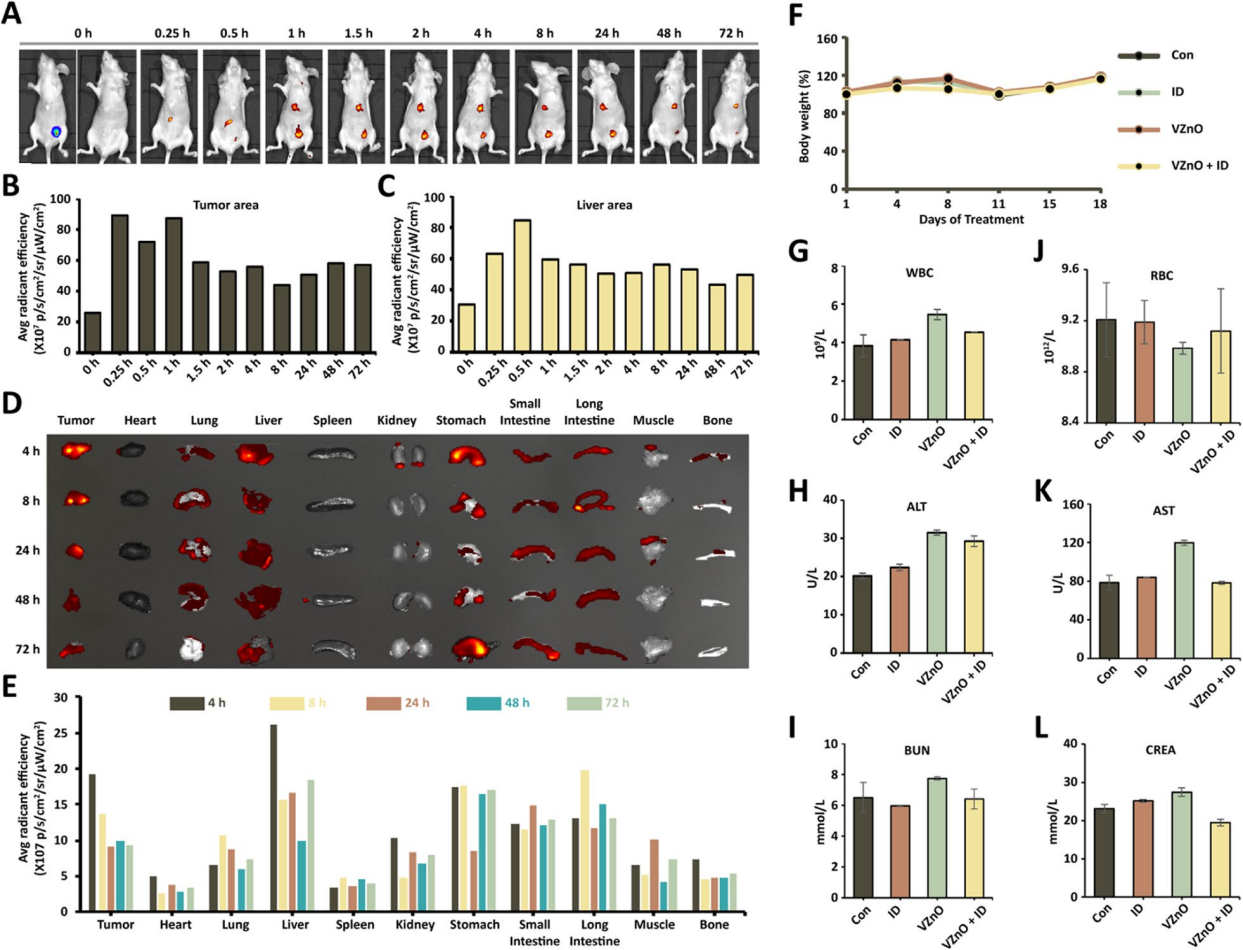
Biodistribution and biosafety of VZnO. **A** In vivo fluorescence imaging of VZnO@FITC in orthotopic CRC tumor after intravenous injection. **B**, **C** The representitive quantitative analysis of the tumor and liver tissues, respectively. **D**, **E** ex vivo imaging and the representitive quantitative analysis. **F** The body weight of Balb/c mice treated with VZnO. **G**–**L** Routine blood, biochemical, liver, and kidney function levels of Balb/c mice on day14

Nanoparticles typically enter tumors through the enhanced osmotic and retention (EPR) effect, through which the nanomaterial passes through openings between or within the endothelial cells of the blood vessels and exudes into the gaps [51, 52]. According to our previous study results, H_2_S in CRC specificity significantly increased (Fig. 1B) [15]. Thus, we expect that VZnO would improve CRC target efficacy owing to excellent specificity. We have detected H_2_S content in tumors in the orthotopic CRC model. To further demonstrate the distribution of the material in the body, we intravenously injected VZnO@FITC into the Balb/c mice, which were examined by an in vivo imaging system. The fluorescence signals in the tumor were detected at 0.5 h after injection of VZnO@FITC, reached a peak at 4 h, and then gradually decreased to the basal level at 48 h (Fig. 4A–C). We collected tissues and performed in vitro fluorescence imaging for further verification. Compared with most normal tissues, except the liver, the tumor tissues had the highest uptake of VZnO@FITC, as evidenced by the fluorescence signals of major organs at various time points in the biodistribution study (Fig. 4D, E).

### Biosafety of VZnO

ZnO nanoparticles are widely utilized for different diseases [43]. At present, the biological toxicity of nano-sized zinc oxide has not been concluded. As nanoparticles' fundamental material properties, particle size is one of the main factors affecting their reactivity and cytotoxicity [53]. To examine the potential toxicity, Balb/c mice were treated with VZnO (50 mg/kg) and ID (50 mg/kg), twice a week for 4 weeks. Administration of treatment by these dosing regimens causes no apparent systemic toxicity as assessed by body weight (Fig. 4F). For example, no significant changes were observed in the counts of red or white blood cells in treated versus control mice. The number of ALT and AST were slightly increased in VZnO-treated mice compared with the control. VZnO also had no significant effect on creatinine, blood urea nitrogen (Fig. 4G). Altogether, VZnO is well tolerated in immune-competent mice.

### Anti-tumor activity of VZnO

**Fig. 5:**
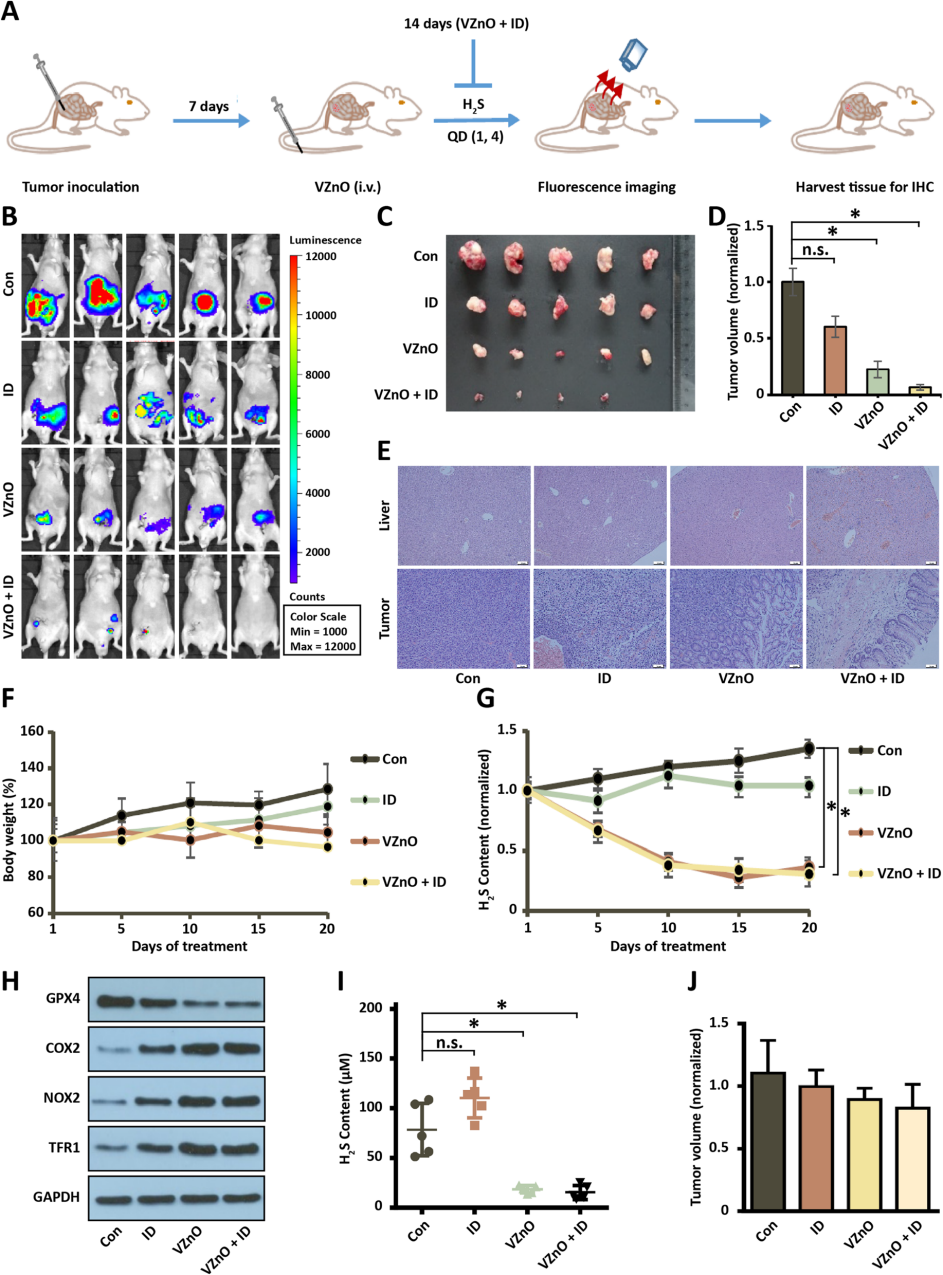
Anti-tumor activity of VZnO in orthotopic CRC model. **A** Experimental timeline of the orthotopic CRC model. **B** Bioluminescence imaging of orthotopic colon cancer mouse injected with different treatments on day 14. **C**, **D** Photograph and volume of the tumors with different treatments on day 14. **E** Representative H&E stained of liver and tumor tissue on day 14. **F** Body weights of orthotopic colon cancer model. **G** The continuously H_2_S production in mouse model. **H** Representative GPX4, COX2, NOX1 and TFR1 protein expression by Western Blot in tumor on day 14. Membranes were re-probed for GAPDH expression to show that similar amounts of protein were loaded in each lane. **I)** H_2_S content measured on day 14. **J** The volume of the tumors in the orthotopic breast model on day 14. (*P < 0.05), (n.s.: No significant difference, *P* value > 0.05)

We next evaluated the therapeutic potential of VZnO (50 mg/kg) and ID (50 mg/kg) using the orthotopic CRC model. The anticancer efficacy of VZnO and ID was evaluated in the orthotopic CRC model by monitoring the tumor burden. Bioluminescence imaging was used to assess the therapeutic efficacy in real-time and non-invasively (Fig. 5A). Because CT26 express luciferases, the bioluminescent intensity of the cells can be used to qualify the survival of the cancer cells. Nude mice bearing CRC were administered PBS, VZnO and ID, respectively. The bioluminescent intensities from the animals were quantified. Among the treatment groups, the combination therapy of VZnO and ID achieved better control of tumor growth, as evidenced by the lowest luciferase activities observed at the end of treatment (day 14) (Fig. 5B–D) and body weight (Fig. 5F). However, VZnO and ID lacked a specific antitumor effect in the orthotopic breast model (low hydrogen sulfide content) (Fig. 5J, Additional file 1: Fig. S8). Immunohistochemistry staining of tumors show combination therapy had the lowest tumor cell density, and no significant abnormalities were found in the liver, lung, spleen, and heart (H&E staining) (Fig. 5E, Additional file 1: Fig. S9).

To evaluate the changes within the tumor after treatment by VZnO and ID. Comparison of tumor tissue in CRC model treated with or without VZnO and ID revealed the selective up-regulation of the ferroptosis-associated protein COX2, NOX1 and TFR1 and down-regulation of GPX4 (Fig. 5H), consistent with previous cell experiments. Following, we proved the content of H_2_S is declined in tumor of CRC model treated with VZnO and ID (Fig. 5I), and the concentration of H_2_S has been reduced to a minimum after about four treatments (Fig. 5G).

### Transcriptome analysis in VZnO-treated HCT116

**Fig. 6:**
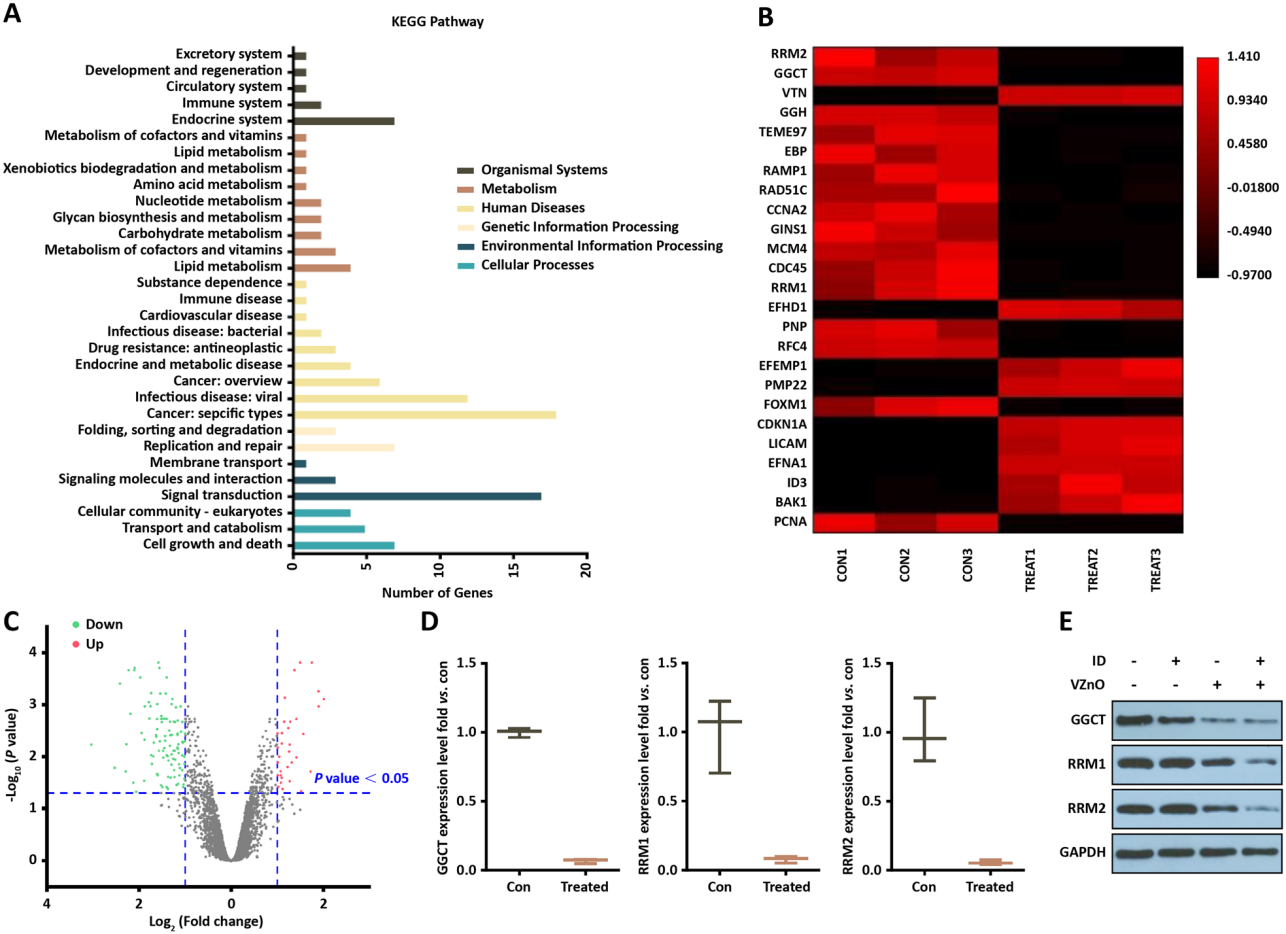
Transcriptome analysis in VZnO-treated HCT116. **A** KEGG pathway analysis based on the RNA-seq results in HCT116. **B** Representative heatmap of gene expression levels. **C** Representative scatter plot of 140 significant genes (33 unregulated genes marked in red and 107 downregulated genes marked in green) for Treat vs. Con. **D** mRNA levels of GGCT, RRM1 and RRM2 by RNA-seq. **E** Representative Western Blot result of GGCT, RRM1 and RRM2. Membranes were re-probed for GAPDH expression to show that similar amounts of protein were loaded in each lane

When ferroptosis are known to mark critical aspects of tumor therapy, global changes could have broad consequences in transcriptomics. To further explore the underlying molecular mechanisms of ferroptosis activated after treatment, the RNA-seq based transcriptome analysis to be used as estimated the transcriptome changes in HCT116 among the control and VZnO/ID groups. There were 33 genes upregulated and 107 genes downregulated in the VZnO/ID-treat group compared with the control group (Fig. 6A, C). Kyoto Encyclopedia of Genes and Genomes (KEGG) pathway analysis showed that these genes were related to cellular motility, growth and death, etc. Among these genes, we found gamma-glutamylcyclotransferase (GGCT), may play a critical role in glutathione homeostasis, whose expression was strongly downregulated after treatment (Fig. 6B, D). Moreover, ribonucleotide reductase M1 (RRM1) and ribonucleotide reductase M2 (RRM2) are also involved in glutathione metabolic pathways according to KEGG pathway analysis (Fig. 6D). And the results were verified by Western Blot (Fig. 6E).

## Conclusion

In summary, we have successfully developed an H_2_S-responsive VZnO nanoplatform for CRC therapy. As a recognized desulfurized, ZnO exhibits outstanding H_2_S scavenging ability and could effectively reduce the H_2_S content in the colorectum. The VZnO nanoparticles can be used as tumor intracellular H_2_S scavengers to induce cell death via ferroptosis with ID. Importantly, VZnO reacts with H_2_S in tumors and efficiently consumes GSH in tumor cells. We found that the tumor cell death was driven by lipid peroxidation, called ferroptosis, after the VZnO reduced the GSH level. We offer evidence linking targeted H_2_S discovery in CRC with ferroptosis, implicating H_2_S is a therapeutic target for CRC. These results may provide a rationale for considering H_2_S clearance as a means to be implicated in CRC. The research is limited, but it does show some promising future therapeutic targets for tumors. At the same time, we also proved that VZnO aggregate at tumor sites in vivo and have good biosafety to immunocompetent Mice. Concordantly, we found that systemic depletion of H_2_S by VZnO in adult mice caused no overt toxicity. This is indicated that VZnO has good biosafety. This therapeutic target warrants further investigation.

Our study has several important implications. First, it provides an example that nanomaterial technology can turn an essentially undruggable target such as H_2_S into a druggable target, thus paving the way for the many human diseases in which H_2_S plays a critical role. Second, it proposed the significance of H_2_S in the tumor and suggested that excessive H_2_S in tumors may be self-protective and harmful to surrounding normal tissues. Third, we performed a biosafety-related toxicological pathology analysis to ensure that the combination of VZnO therapy targeting tumors has low toxicity and high safety. In summary, we provide robust evidence supporting VZnO as a selective and efficient H_2_S scavenger for colorectal cancer treatment. Our research provides a solid foundation for the clinical development of H_2_S scavenging agents for H_2_S-relative human cancer and other human diseases. We believe that this design approach will advance the development of materials for tumor-specific therapy with less toxicity and side effects on normal tissues.

## Supplementary Information

**Additional file 1:** Supplementary data to this article can be found online including Materials, additional Experimental Methods of in vitro and in vivo.

## Data Availability

All data generated or analyzed during this study are included in this manuscript and its Additional file 1.
