# Supplementary material for: Zinc oxide nanosphere for hydrogen sulfide scavenging and ferroptosis of colorectal cancer
Source: J Nanobiotechnology. 2021 Nov 27;19:392. doi: 10.1186/s12951-021-01069-y (PMC8626909; doi:10.1186/s12951-021-01069-y)
Supplement: Supplementary file 1 — Additional file 1: Supplementary data to this article can be found online including Materials, additional Experimental Methods of in vitro and in vivo. [file 12951_2021_1069_MOESM1_ESM.docx]

Supplementary information

Zinc Oxide Nanosphere for Hydrogen Sulfide Scavenging and Ferroptosis of Colorectal Cancer

Xiang Pan^1,4#^, Yuchen Qi^2#^, Zhen Du^2*^, Jian He^2^, Sheng Yao^4,5^, Wei Lu^1^, Kefeng Ding^1,3*^, Min Zhou^1,2,3,4*^

1. Department of Colorectal Surgery and Oncology, Key Laboratory of Cancer Prevention and Intervention, Ministry of Education, The Second Affiliated Hospital, Zhejiang University School of Medicine, Hangzhou, Zhejiang, China

2. Institute of Translational Medicine, Zhejiang University, Hangzhou, 310029, China

3. State Key Laboratory of Modern Optical Instrumentations, Zhejiang University, Hangzhou, 310058, China

4. Cancer Center, Zhejiang University, Hangzhou, Zhejiang 310058, China

5. Laboratory of Gastroenterology, The Second Affiliated Hospital, Zhejiang University School of Medicine, Hangzhou, 310029, China

^#^ These authors contributed equally to this work.

**^*^ Address correspondence to**

zhoum@zju.edu.cn (Min Zhou);

dingkefeng@zju.edu.cn (Kefeng Ding);

duzhen90@ zju.edu.cn (Zhen Du)


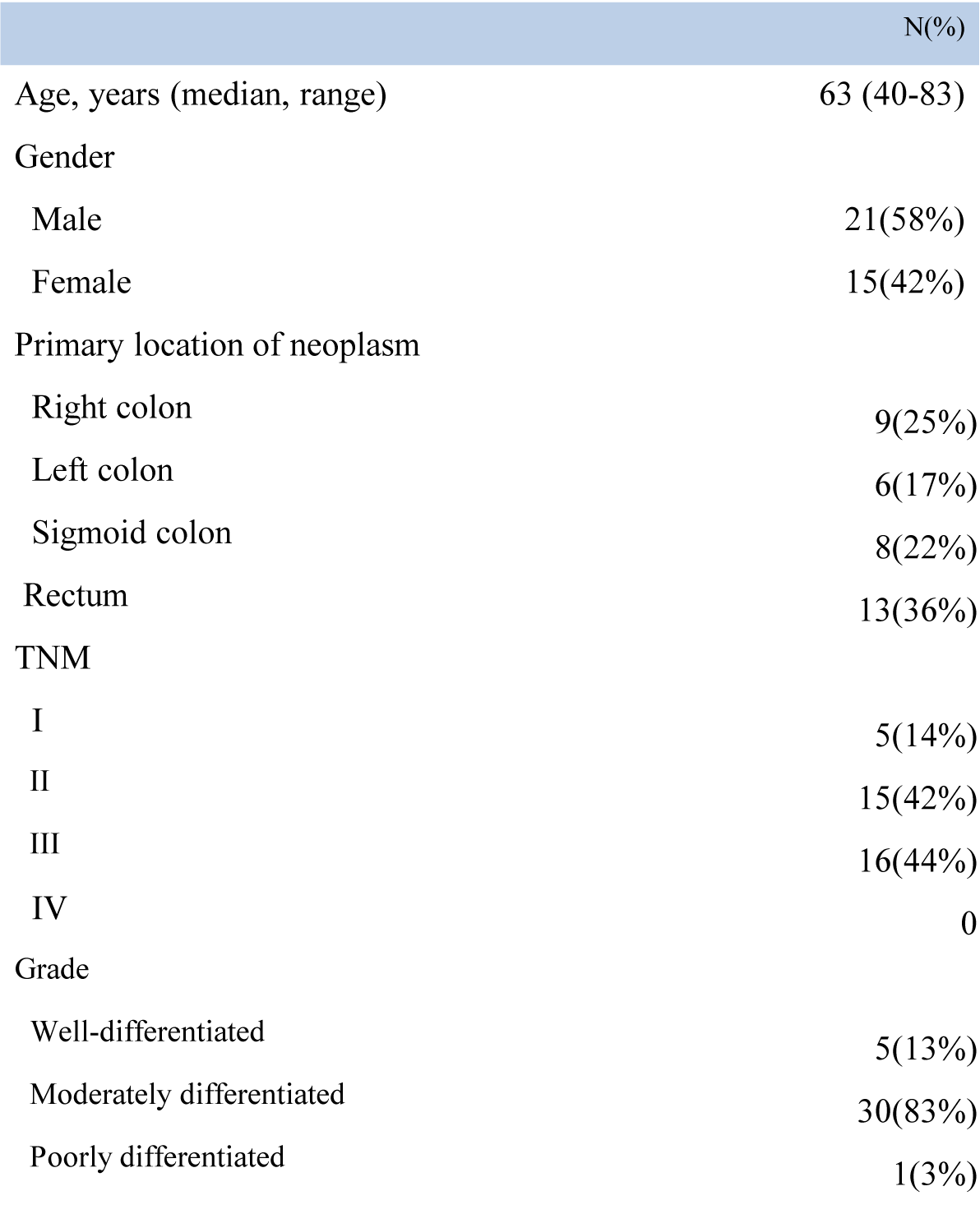


**Table S1.** Demographic, clinical and pathological data of 36 patients with colon cancer.

**
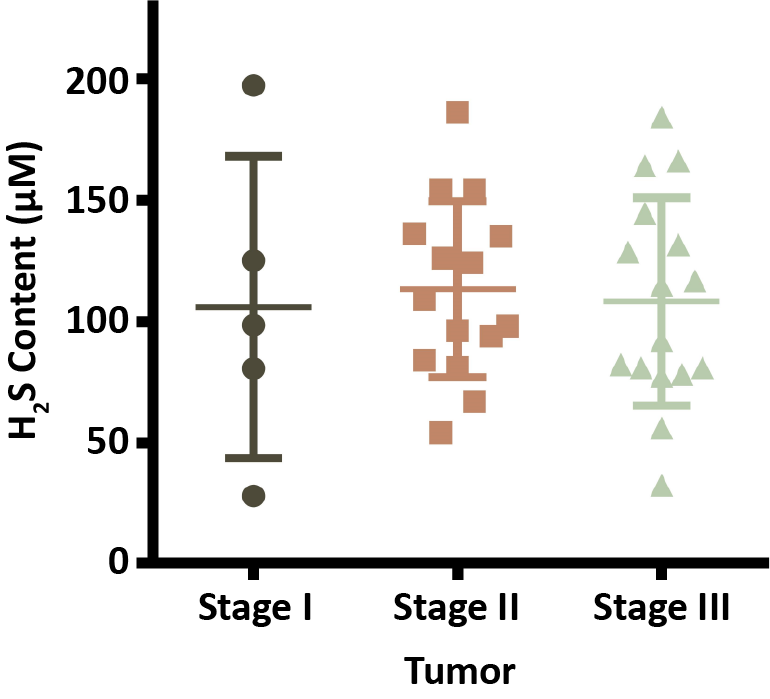
**

**Figure S1.** The H_2_S content in human colon cancer at different stage.


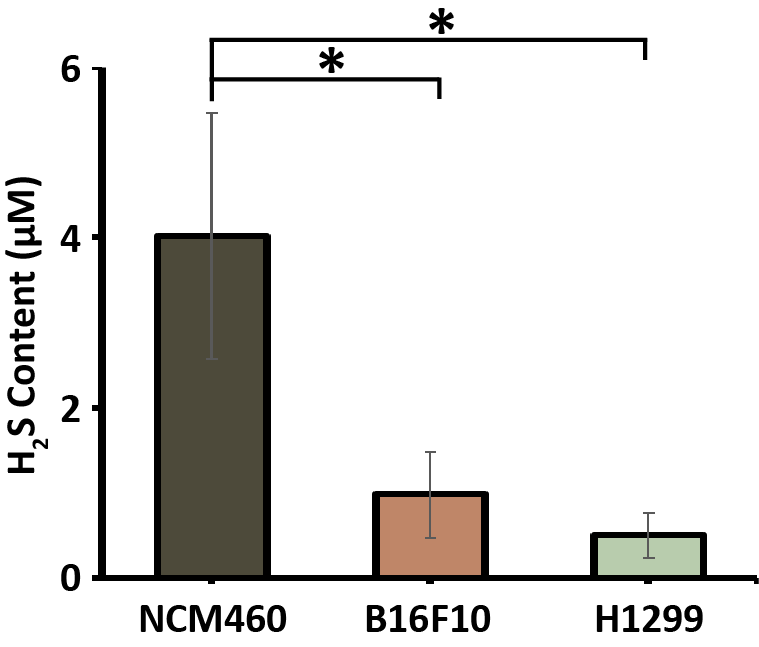


**Figure S2.** The H_2_S content of NCM460 (normal human colon mucosal epithelial cell), B16F10 (melanoma) and H1299 (non-small cell lung cancer cell), respectively.

**
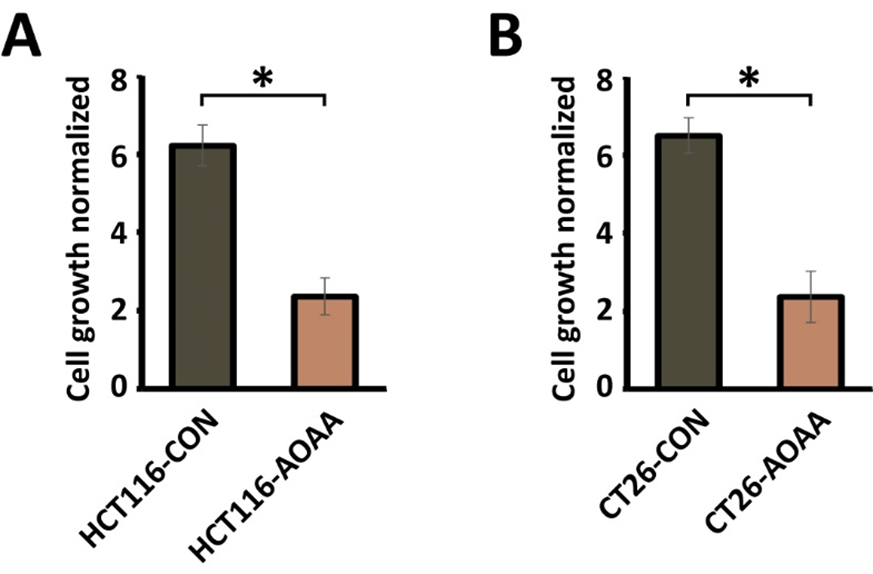
**

**Figure S3. A)** The cell growth of the HCT116 cell treated with AOAA for 24 h (*P < 0.05). **B)** The cell growth of the CT26 cell treated with AOAA for 24 h (*P < 0.05).

**
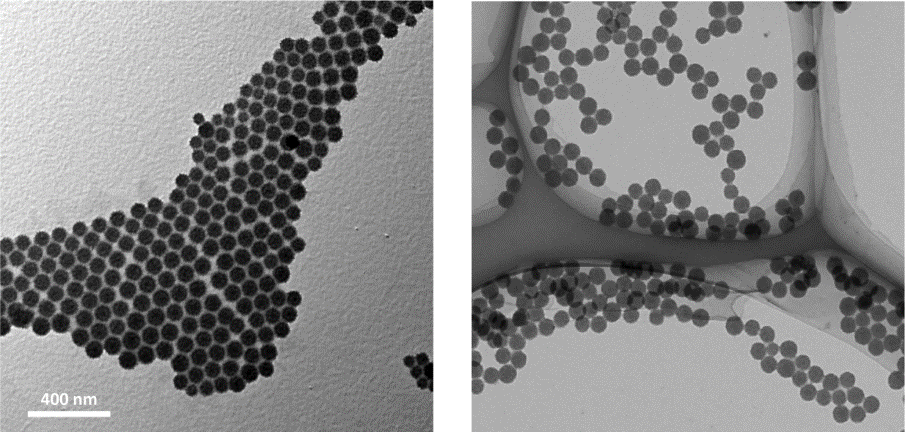
**

**Figure S4.** Transmission electron microscopy (TEM) images of the virus-like silica nanoparticles (VMSN).

**Figure S5.** Fluorescence spectrum of the VZnO@FITC nanoparticles.


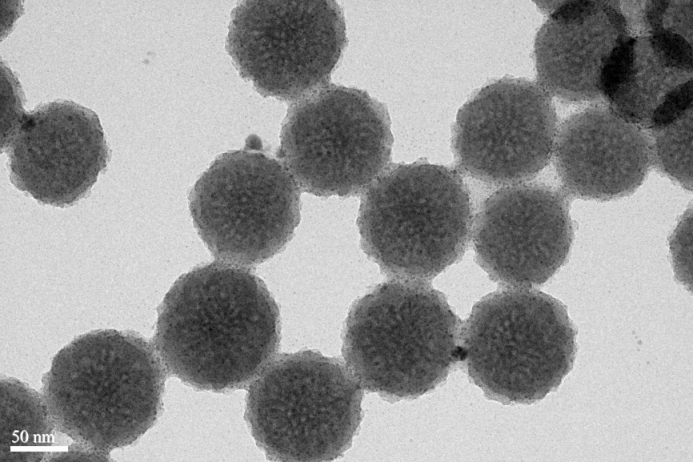


**Figure S6.** TEM image of the VZnO nanoparticles after a short time sulfidation reaction.


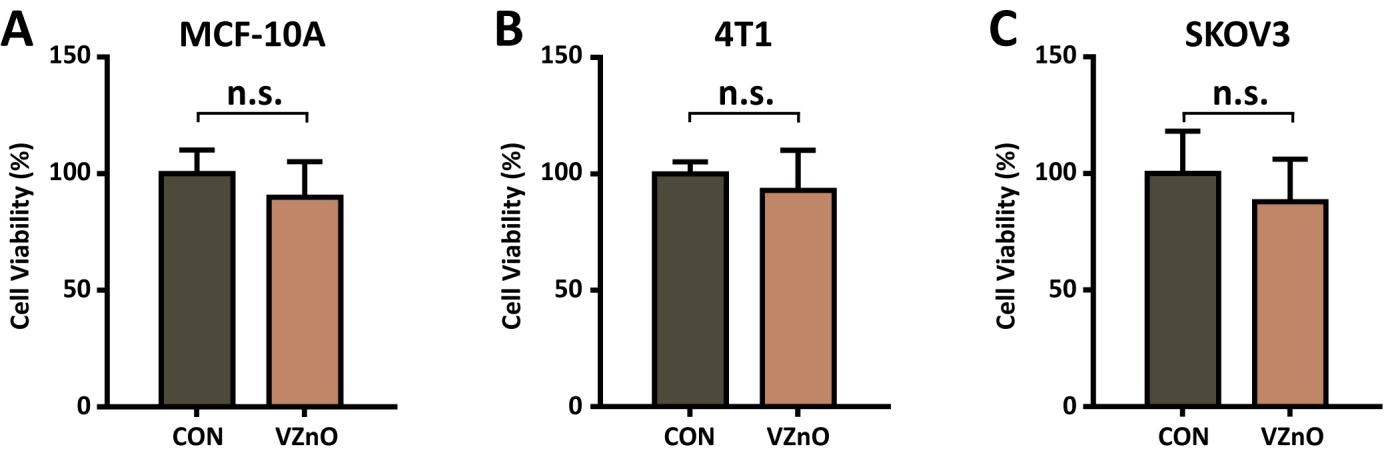


**Figure S7. A)** Cell viability by MTT assay in MCF-10A cell after VZnO treated. **B)** Cell viability by MTT assay in 4T1 cell after VZnO treated. **C)** Cell viability by MTT assay in SKOV3 cell after VZnO treated.

**
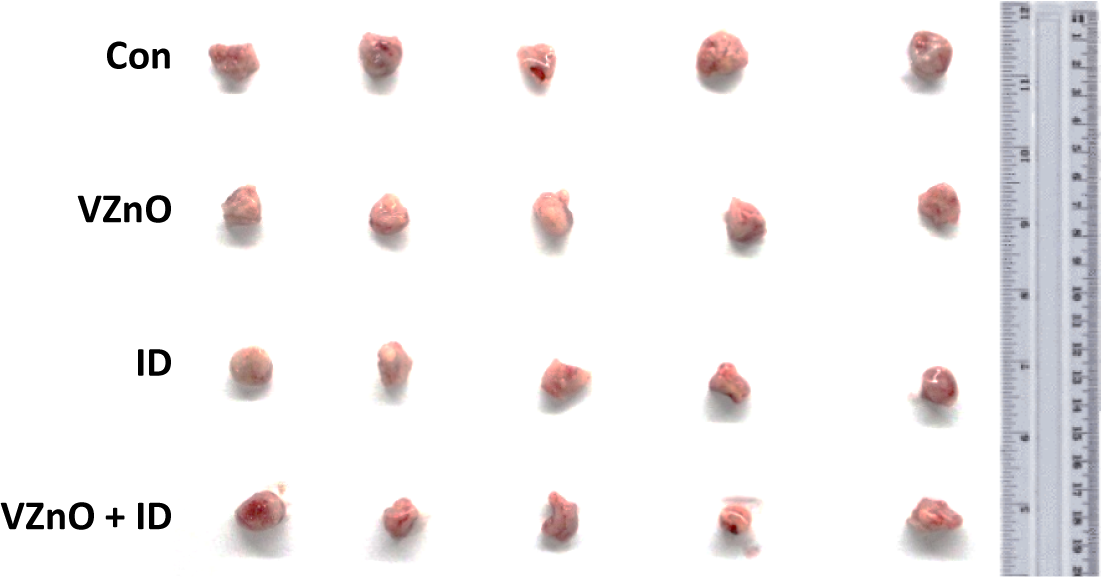
**

**Figure S8.** Photograph and volume of the orthotopic breast model tumors with different treatments on day 14.

**
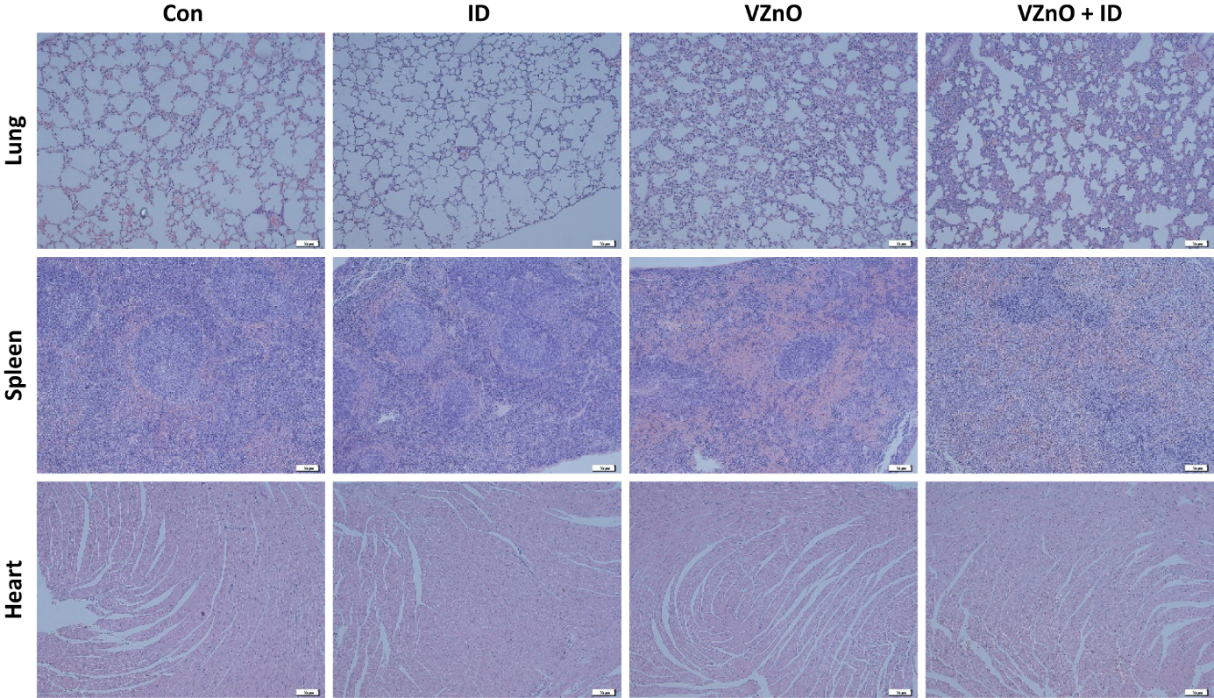
**

**Figure S9.** Representative H&E stained of lung, spleen and heart tissue in the orthotopic colorectal cancer model treated with VZnO on day 14.
